# Hepatocyte Growth Factor Genetic Variations and Primary Angle-Closure Glaucoma in the Han Chinese Population

**DOI:** 10.1371/journal.pone.0060950

**Published:** 2013-04-09

**Authors:** Zhengxuan Jiang, Kun Liang, Biqing Ding, Wei Tan, Jing Wang, Yunxia Lu, Yuxin Xu, Liming Tao

**Affiliations:** 1 Department of Ophthalmology, The Second Affiliated Hospital of Anhui Medical University, Hefei, Anhui, China; 2 Department of Ophthalmology, The First People’s Hospital of Zunyi, the Third Affiliated Hospital of Zunyi Medical University, Zunyi, Guizhou, China; 3 Department of Biochemistry and Molecular Biology, Anhui Medical University, Hefei, Anhui, China; University of Rochester, United States of America

## Abstract

**Purpose:**

The aim of this study is to examine whether or not hepatocyte growth factor (HGF) genetic variations are associated with susceptibility to primary angle-closure glaucoma (PACG) in the Han Chinese population.

**Methods:**

Three single-nucleotide polymorphisms (SNPs)–rs5745718, rs17427817, and rs3735520–in the HGF gene were genotyped in 238 adult patients with PACG and 287 age-, sex-, and ethnically matched healthy controls by using a polymerase chain reaction restriction fragment length polymorphism assay. Data was analyzed by χ^2^ analysis.

**Results:**

The three tested analyzed polymorphisms in the HGF gene were in Hardy-Weinberg equilibrium, in all the subjects. The frequencies of the genotype and allele of rs5745718 and rs1742817 in the HGF gene were significantly different between the PACG patients and the controls. On one hand, the frequencies of the CC genotype and C allele of rs5745718 were significantly decreased in PACG patients compared with controls (P_c_ = 1.40×10^−3^; P_c_ = 3.21×10^−4^, respectively); however, on the other hand, significantly decreased frequencies of the GG genotype and the G allele of rs17427817 were observed in PACG patients compared with the controls (P_c_ = 0.006,; P_c_ = 6.06×10^−4^, respectively). A comparison of the distributions of the genotypes and alleles of rs3735520 showed no statistically significant differences between the PACG patients and the controls (p_c_>0.05). The haplotype analysis results showed that the CGC haplotype frequency was significantly decreased in the patients with PACG compared with the controls (p_c_<0.001). No difference was detected between the patients and the controls with regard to the other haplotypes.

**Conclusions:**

Our study suggests that rs5745718 and rs17427817 are associated with a decreased risk of PACG in the Chinese Han population. The CGC haplotype was demonstrated to possibly play a protective role against PACG in this population.

## Introduction

Glaucoma is the second largest cause of blindness worldwide after cataracts. It is estimated that 70 million people suffer from glaucoma globally, and there will be 79.6 million people with glaucoma and about 11.2 million patients with bilateral blindness by 2020 [1]. Primary angle-closure glaucoma (PACG) has relatively high visual morbidity rates and accounts for half of all blind glaucoma patients in the Chinese Han population [2]. Epidemiological studies have revealed that the majority of patients with PACG live in Asian regions, especially in China [3], [4].

PACG is characterized by a diverse spectrum of clinical manifestations including a shallow anterior chamber [5], increased thickness of the lens [4], and a short axial length [6], often accompanied by hypermetropic refraction error [7], [8]. The etiology of PACG remains largely unknown and is thought to be a multifactorial disease. It is assumed that a genetic predisposition as well as environmental elements are involved in the development of this disease [9]. Genetic factors play an important role in the occurrence of PACG. Previous studies have revealed that the risk of developing PACG is much higher in families in which first-degree relatives have the disease [10]. Several candidate genes are associated with PACG in different populations, including the matrix metalloproteinase-9 gene [11], [12], myocilin [13], optineurin [14], and tumor necrosis factor-α [15]. However, these genes only partly explain the genetic predisposition to PACG, and more research is needed to determine the causative genes of this disease.

Hepatocyte growth factor (HGF) was thought to play an important role in the emmetropization process of the eye [16], and several single nucleotide polymorphisms (SNPs) of the HGF gene were firstly reported to be associated with hyperopia. As angle-closure glaucoma and hyperopia share the same feature of a short axial length, the HGF gene may be also a risk factor of PACG [17]. A recent study revealed that four SNPs of the HGF gene are associated with susceptibility to PACG in the Nepalese population [18]. There is much evidence to support the fact that genetic heterogeneity exists in different ethnic cohorts, even in the same population which comes from a different region. Therefore, this data prompted us to search for a relationship between SNPs of the HGF gene with a risk occurrence of PACG. We designed this case-control study and examined whether or not HGF polymorphisms were associated with susceptibility to PACG in the Han Chinese population.

## Methods

### Subjects

A total of 238 patients with PACG and 287 age-, sex-, and ethnically matched healthy controls were recruited from the Second Affiliated Hospital of Anhui Medical University (Anhui, People’s Republic of China). Both the patients and healthy controls underwent a full ophthalmic examination, which included a visual acuity test, a slit-lamp examination of the anterior chamber, a gonioscopy, the measurement of central corneal thickness and intraocular pressure, a fundus examination with special attention to optic disc parameters, and a visual field test. A total of 238 patients were selected for the PACG group according to the following diagnostic criteria: the presence of glaucomatous optic neuropathy with a cup:disc ratio ≥0.7, peripheral visual loss, an intraocular pressure of more than 21 mmHg, and the presence of at least 180 degrees of closed angle in which the trabecular meshwork was not visible on a gonioscopy. Patients with secondary angle-closure glaucoma, which was caused by uveitis, trauma, or lens subluxation, were excluded. The healthy individuals came from the same geographical regions as the PACG patients, and the controls were age-, sex- and ethnically matched with the PACG patients. They were enrolled into the control group according to the following criteria: (1) none of the PACG symptoms or signs mentioned above; (2) no glaucoma history and familial glaucoma history; and (3) no ophthalmic diseases other than cataracts. The clinical characteristics of these subjects are shown in Table 1. The study was approved by the local institutional ethnics committee of the Second Affiliated Hospital of Anhui Medical University and met the tenets of the Declaration of Helsinki. Informed consent was obtained from all of the subjects. We obtained 5 ml of peripheral blood from each individual.

**Table 1:** Clinical characteristics of study participants.

Clinical features	PACG patients	Controls
Age (mean±SD)	56.2±10.7	58.4±11.2
Number	238	287
Sex (% male)	50.4%.	52.3%
IOP in mmHg (mean±SD)	24.6±8.7	12.3±7.2
C/D (mean±SD)	0.72±0.11	0.22±0.09

### DNA Extraction and SNP Selection and Genotyping

Venous blood samples were obtained from the PACG patients and the controls in EDTA tubes and kept at −70°C until use. Genomic DNA was extracted from the peripheral venous blood using the QiaAmp DNA Blood Mini Kit (Qiagen, Hilden, Germany) according to the manufacturer’s instructions.

As reported in earlier studies, since there is an association between HGF polymorphisms, and PACG and hyperopia in other ethnic groups [16], [18], we selected rs17427817, rs5745718, and rs3735520 as candidate SNPs. Amplification of the target DNA in the HGF gene was analyzed by polymerase chain reaction (PCR) using the primers presented in Table 2. The PCR restriction fragments length polymorphism method was employed to determine the genotypes of the tested SNPs. Each PCR reactive solution consisted of the following: 5 µl Premix Taq (Ex Taq Version; TaKaRa Biotechnology Co. Ltd., Dalian, China), 20 pmol primers, and 0.2 µg genomic DNA. The conditions were as follows: initial denaturation at 95°C for 5 minutes followed by 38 cycles of denaturation at 94°C for 30 seconds, annealing at different temperatures (54°C for rs17427817 and rs5745718, 56°C for rs3735520) for 30 seconds, extension at 72°C for 30 seconds, and a final extension at 72°C for 5 minutes. The PCR products of the rs17427817, rs5745718, and rs3735520 polymorphisms were respectively digested with 2 U of NlaIV (MBI Fermentas, Burlington, ON, Canada), BssECI (MBI Fermentas), and BglII (Promega, Madison, WI, USA) restriction enzymes (Table 2) in a 10-µl reaction volume overnight. The digestion products were visualized on a 3.5% agarose gel and stained with GoldView (SBS Genetech, Beijing, China).

**Table 2:** Primers of HGF SNPs and restriction enzymes used for FRLP analysis.

SNP	Primers	Restriction enzyme
rs17427817	5′-TCCCTCGGGATTGAGAACAGTG-3′	NlaIV
	5′-TTTGGAGGTCTTTACTACTT-3′	
rs5745718	5′-CTACAAAGAACCTACAACACAA-3′	BssECI
	5′-TCATCTTACTGAACTTGTTT-3′	
rs3735520	5′-ACCCTAATAAAGCACAAGA-3′	BglII
	5′-CCTGCCTGATAAGTCCCTGAG-3′	

### Statistical Analysis

The differences in age and gender between the cases and the controls were assessed by t-test and χ^2^ test, respectively. The Hardy-Weinberg equilibrium was tested using the χ^2^ test. The significance of the differences in the allele and genotype distribution between the patients and the controls was evaluated with the use of the χ^2^ test performed by using SPSS (version 11.0, SPSS, Inc., Chicago, IL, USA). When the number of genotypes or alleles was fewer than 5 counts, the Fisher’s exact test was used. The P-values were corrected (Pc) with the Bonferroni correction by multiple comparison with the number of analyses performed. A P-value of less than 0.05 was considered statistically significant.

## Results

The patients in the PACG cohort numbered 238 and included 93 male and 114 female subjects. The average age of the PACG patients was 61.3±10.2 years. The healthy controls cohort included 287 subjects (150 male, 137 female) with a mean age of 64.7±8.7 years. The demographic characteristics between the PACG cases and the healthy controls were similar. There was no significant difference between the cases and the controls with respect to age and gender. The clinical features of the patients with PACG and the healthy controls are summarized in Table 2.

Three single-nucleotide polymorphisms of the HGF gene were successfully genotyped in the 238 PACG patients and 287 healthy controls. The observed genotype frequencies of the three tested SNPs in this study were in agreement with the Hardy-Weinberg equilibrium in both the cases and the controls. The genotype and allele frequency and odds ratio of the tested HGF polymorphisms in the study and control group are displayed in Table 3. The frequencies of the genotype and the allele of rs5745718 and rs1742817 in the HGF gene were significantly different between the PACG patients and the controls. On one hand, the frequencies of the CC genotype and C allele of rs5745718 were significantly decreased in patients with PACG compared with controls (χ^2^ = 13.54, Pc = 1.40×10^−3^, OR 0.46, 95% CI 0.30–0.69; χ^2^ = 15.02, Pc = 3.21×10^−4^, OR 0.48, 95% CI 0.33–0.69; respectively). On the other hand, significantly decreased frequencies of the GG genotype and the G allele of rs17427817 were observed in the PACG patients compared with the controls (χ^2^ = 10.87, Pc = 0.006, OR 0.52, 95% CI 0.35–0.77 and χ^2^ = 13.81, Pc = 6.06×10^−4^, OR 0.52, 95% CI 0.37–0.74, respectively). A comparison of the distribution of genotypes and alleles of rs3735520 showed no statistically significant differences between the PACG patients and the controls (P>0.05).

**Table 3:** Frequencies of alleles and genotypes of HGF polymorphisms in PACG patients and controls.

SNP	Genotype/Allele	PACG(N = 238)	Controls(N = 287)	χ^2^	P-value	Pc	OR(95% CI)
rs5745718	AA	9 (3.8%)	4 (1.4%)	3.072	0.095	NS	2.78 (0.85–9.14)
	AC	61 (25.6%)	42 (14.6%)	9.976	0.002	0.012	2.01 (1.30–3.12)
	CC	168 (70.6%)	241 (84.0%)	13.539	2.34×10^−4^	1.40×10^−3^	0.46 (0.30–0.69)
	A	79 (16.6%)	50 (8.7%)	15.016	1.07×10^−4^	3.21×10^−4^	2.09 (1.43–3.04)
	C	397 (83.4%)	524 (91.3%)	15.016	1.07×10^−4^	3.21×10^−4^	0.48 (0.33–0.69)
rs17427817	CC	11 (4.6%)	4 (1.4%)	5.082	0.024	NS	3.51 (1.10–11.2)
	CG	68 (28.6%)	54 (18.8%)	6.942	0.009	NS	1.73 (1.15–2.60)
	GG	159 (66.8%)	229 (79.8%)	10.873	0.001	0.006	0.52 (0.35–0.77)
	C	90 (18.9%)	62 (10.8%)	13.811	2.02×10^−4^	6.06×10^−4^	1.93 (1.36–2.73)
	G	386 (81.1%)	512 (89.2%)	13.811	2.02×10^−4^	6.06×10^−4^	0.52 (0.37–0.74)
rs3735520	TT	92 (38.7%)	95 (33.1%)	1.750	0.186	NS	1.27 (0.89–1.82)
	TC	112 (47.1%)	139 (48.4%)	0.098	0.754	NS	0.95 (0.67–1.34)
	CC	34 (14.2%)	53 (18.5%)	1.645	0.200	NS	0.74 (0.46–1.18)
	T	296 (62.2%)	329 (57.3%)	2.559	0.110	NS	1.23 (0.96–1.57)
	C	180(37.8%)	245(42.7%)	2.559	0.110	NS	0.82 (0.64–1.05)

PACG = primary angle-closure glaucoma; OR = odds ratio; 95% CI = 95% confidence interval; Pc = Bonferroni corrected P-value; NS = not significant.

A haplotype analysis was performed by SHEsis platform [19], and the results are shown in Table 4. The CGC haplotype frequency was significantly decreased in patients with PACG compared with the controls (Pc<0.001, OR 0.59, 95% CI 0.45–0.77). No differences were detected between the patients and the controls with regard to the other haplotypes.

**Table 4:** Results of haplotypes formed by rs5745718, rs17427817, and rs3735520 at HGF locus in PACG patients and controls in the Chinese Han population.

Haplotype	Case, Control Ratios	Pc	OR (95% CI)
AGC	0.058, 0.027	NS	–
AGT	0.105, 0.060	NS	–
CCC	0.037, 0.017	NS	–
CCT	0.148, 0.113	NS	–
CGC	0.283, 0.383	0.006	0.64 (0.49–0.83)
CGT	0.365, 0.403	NS	–

Pc = Bonferroni corrected P; OR = odds ratio; 95% CI = 95% confidence interval.

## Discussion

Glaucoma is a neurodegenerative disease, which causes a progressive degeneration of the retinal ganglion cells in the optic disc or retinal nerve fiber. In this study, our results showed that the two analysed SNPs of HGF, rs17427817 and rs5745718, were associated with a decreased risk for PACG. A haplotype analysis revealed that people with the CGC haplotype are less susceptible to PACG. Additionally, the SNP rs3733520 in the HGF gene was not associated with PACG in this study.

The HGF protein has been shown to be involved in causing disruption to the emmetropization process within the eye and stimulating the growth and migration of many tissues of the eye [20]–[22]. Recent studies have found that some SNPs of the HGF gene were associated with susceptibility to hyperopia [16]. Several SNPs of the HGF gene were also identified as being associated with PACG in the Nepalese population [18].

Several strategies can be used to select a candidate gene in the study of genetic susceptibility to a certain disease. For example, one gene can be selected for study according to the functions that are involved in the pathogenesis of the relevant disease [23]. In this study, we selected the HGF gene as a candidate gene based on number of factors. First, earlier studies revealed that the mRNA level of HGF is higher in the rabbit lacrimal gland after corneal injury [24], and the level of HGF protein is significantly increased in the aqueous humour of glaucomatous eyes [25]. These results suggested that the HGF protein might have functional effects in the development of PACG. Second, it has been suggested that the upregulated expression of a certain gene may be associated with certain SNP alleles in its gene. The polymorphisms of the HGF gene have been widely studied in hyperopia in different populations. There is evidence to support the hypothesis that PACG and hyperopia have potentially similar cellular events in the pathogenesis of the two diseases [17]. Third, primary angle-closure glaucoma is one of the leading causes of visual morbidity in China. Based on the functional studies of HGF, the genetic factor in this disease, and the association between the HGF gene and PACG in other populations [18], we designed a case-control association study to clarify whether or not polymorphisms of the HGF gene were associated with PACG in the Chinese Han population. As with the other association studies, many factors may influence the association results. We strictly selected patients who had been diagnosed with PACG according to international criteria. The subjects in the study were all from the Chinese Han population, and the healthy controls were age- and sex-matched with the PACG patients in order to avoid ethnic confounding.

Since one candidate gene has numerous SNPs and only a few of them may be involved in the development of the disease, it is extremely important to select the appropriate SNPs [26]. We selected rs17427817, rs5745718, and rs3735520 as the candidate SNPs based on the fact that these SNPs in the HGF gene were reported to be associated with hyperopia in the Chinese Han population and PACG in other populations [16], [18]. On one hand, our study found two SNPs in the HGF gene were significantly associated with PACG in the Chinese Han population. The rs17427817 G allele and GG genotype were associated with decreased susceptibility to PACG in the Chinese Han population, CC genotype and C allele of the rs5745718 was also associated with a reduction in the risk of PACG. These results were consistent with previous studies which reported that rs17427817 and rs5745718 were associated with PACG in the Nepalese population and which reported that rs5745718 was associated with hyperopia in Caucasians [16], [18]. This data revealed that the HGF gene may be a common susceptibility gene of PACG in the different populations [18]. On the other hand,this study failed to find an association between the rs3735520 SNP and PACG, which is consistent with previous findings regarding PACG in the Nepalese population and hyperopia in the Han Chinese population [16], [18]. However, the rs3735520 was reported to be associated with mild to moderate myopia in Caucasian population and keratoconus in Australian. Interesting [27], [28], the SNP was identified to be associated with longer vitreous chamber depth in Chinese population [29]. Taken together, these results indicate that PACG and hyperopia possibly share a common pathway in the development of these diseases.

A haplotype analysis revealed that the haplotype CGC conferred a reduced risk of PACG. It is clear from analysing the results of Table 4 that the CG haplotype formed by the C allele of rs5745718 and the G allele of rs17427817 is a protective haplotype. This result revealed that the C allele of rs5745718 and the G allele of rs17427817 were associated with reducing the risk of PACG, which is consistent with the results in Table 3. The patterns of linkage disequilibrium (LD) of the tested SNPs in this studied samples were compared with that available in the Nepalese population [18], and the results showed no significant difference among them. The LD results of the studies were similar with that in the international HapMap for the Han Chinese population.

Like other candidate gene association studies, there are some limitations in our study. First, the ability to plausibly detect disease susceptibility genes is influenced by the sample group. The size of the patient sample group in our study was relatively small, and the patients were recruited only from the Han Chinese population. The results observed in this study need to be confirmed using larger sample sizes and other ethnic populations. Second, as rs5745718 and rs17427817 polymorphisms are in non-coding regions, their functional roles are unknown, and they may be in a linkage disequilibrium with the causative variants in the HGF gene. Some other SNPs need to be further investigated.

In conclusion, to the best of our knowledge, this is the first association study between HGF gene and PACG in the Chinese Han population. This study has revealed that the CC genotype and C allele of rs5745718 and the GG genotype and G allele of rs174278717 may be associated with a decreased risk of PACG in the Chinese Han population. The CGC haplotype has been demonstrated to possibly play a protective role against PACG in this population.
